# 2,7-Bis(4-acetyl­phen­oxy)naphthalene

**DOI:** 10.1107/S1600536808007496

**Published:** 2008-03-29

**Authors:** Kosuke Nakaema, Masahiro Imaizumi, Keiichi Noguchi, Noriyuki Yonezawa

**Affiliations:** a Department of Organic and Polymer Materials Chemistry, Tokyo University of Agriculture & Technology, Koganei, Tokyo 184-8588, Japan; b Section Manager, Group I, Section III, Functional Chemicals Research Laboratory, Nippon Kayaku Co. Ltd, Shimo 3-chome, Kita-ku, Tokyo 115-0042, Japan; c Instrumentation Analysis Center, Tokyo University of Agriculture & Technology, Koganei, Tokyo 184-8588, Japan

## Abstract

The title compound, C_26_H_20_O_4_, has an asymmetrical conformation at 193 K. The 4-acetyl­phenyl groups are twisted away from the the naphthalene ring system, with one benzene ring turned towards the 1-position of the naphthalene ring and the other benzene ring turned towards the 6-position. The inter­planar angles between the mean planes of the benzene rings and the naphthalene ring system are 68.71 (6) and 74.01 (6)°. The structure displays C—H⋯O hydrogen bonding and π–π stacking inter­actions [centroid–centroid and interplanar distances are 3.5938 (9) and 3.517 Å, respectively].

## Related literature

For related literature, see: Ocak *et al.* (2004 ▶).
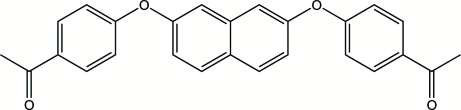


## Experimental

### 

#### Crystal data

C_26_H_20_O_4_
*M* *_r_* = 396.42
Triclinic, 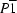

*a* = 5.8691 (2) Å
*b* = 7.9105 (2) Å
*c* = 21.4040 (5) Å
α = 90.322 (2)°
β = 95.534 (2)°
γ = 102.283 (2)°
*V* = 966.11 (5) Å^3^
*Z* = 2
Cu *K*α radiation
μ = 0.74 mm^−1^
*T* = 193 K
0.60 × 0.20 × 0.02 mm

#### Data collection

Rigaku R-AXIS RAPID diffractometer
Absorption correction: numerical (*NUMABS*; Higashi, 1999 ▶) *T*_min_ = 0.792, *T*_max_ = 0.985
17040 measured reflections
3467 independent reflections
2617 reflections with *I* > 2σ(*I*)
*R*_int_ = 0.030

#### Refinement

*R*[*F* ^2^ > 2σ(*F* ^2^)] = 0.043
*wR*(*F* ^2^) = 0.125
*S* = 1.09
3467 reflections
273 parameters
H-atom parameters constrained
Δρ_max_ = 0.19 e Å^−3^
Δρ_min_ = −0.24 e Å^−3^

### 

Data collection: *PROCESS-AUTO* (Rigaku, 1998 ▶); cell refinement: *PROCESS-AUTO*; data reduction: *CrystalStructure* (Rigaku/MSC, 2004 ▶); program(s) used to solve structure: *SIR2004* (Burla, *et al*, 2005 ▶); program(s) used to refine structure: *SHELXL97* (Sheldrick, 2008 ▶); molecular graphics: *ORTEPIII* (Burnett & Johnson, 1996 ▶); software used to prepare material for publication: *SHELXL97*.

## Supplementary Material

Crystal structure: contains datablocks global, I. DOI: 10.1107/S1600536808007496/fl2193sup1.cif

Structure factors: contains datablocks I. DOI: 10.1107/S1600536808007496/fl2193Isup2.hkl

Additional supplementary materials:  crystallographic information; 3D view; checkCIF report

## Figures and Tables

**Table 1:** Hydrogen-bond geometry (Å, °)

*D*—H⋯*A*	*D*—H	H⋯*A*	*D*⋯*A*	*D*—H⋯*A*
C2—H2⋯O2^i^	0.95	2.54	3.448 (2)	160

Symmetry code: (i) 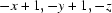.

**Fig. 1.**
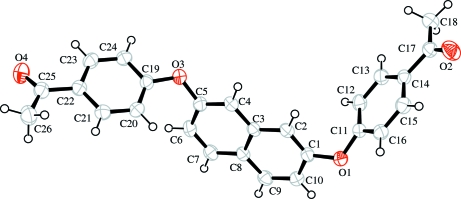
Molecular structure of (I), with the atom-labeling scheme and displacement ellipsoids drawn at the 50% probability level.

**Fig. 2.**
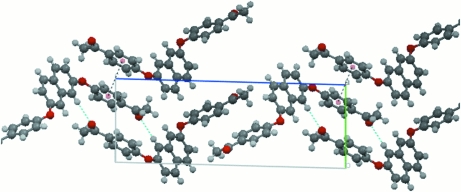
The crystal packing of the title compound, viewed down the a axis. The dashed lines indicate hydrogen bonding (blue dashed line) and π—π stacking interactions (black dashed line).

## supplementary crystallographic information

### Comment

An *ORTEPIII* (Burnett & Johnson, 1996) plot of the molecule (I) is shown in Fig. 1.Considering its two dimensional representation, the molecule could have had *C_2_* symmetry. This is certainly not the case in practice, as the naphthalene moiety forms dihedral angles of 68.71 (6)° and 74.01 (6)° with the best mean planes of the aromatic rings C11—C16 and C19—C24, respectively. The torsion angles between the naphthalene ring and the two benzene rings are -34.0 (2)° [C11—O1—C1—C2], and -132.00 (15)° [C19—O3—C5—C4]. The difference in the two torsion angles between the naphthalene and benzene rings is rather large. This means that one benzene ring (C11—C16) turns to the 1-position, and the other benzene ring (C19—C24) turns to the 6-position rather than the 8-position. This compound has an asymmetrical conformation similar to that of 2,7-bis(3,4-dicyanophenoxy)naphthalene (Ocak *et al.*, 2004).

The crystal packing is stabilized mainly by van der Waals interactions, however there is some π—π stacking and C—H···O intermolecular interactions (Table 1, Fig. 2). The hydrogen bonds between an acetyl hydrogen and the carbonyl oxygen of a neighboring molecule link the molecules into pairs around a center of symmetry that are aligned complementarily in a row forming a polymer-like infinitive ribbon (Fig. 2).

### Experimental

2,7-naphthalenediol (160 mg, 1.0 mmol) and 4-fluoroacetophenone (303 mg, 2.2 mmol) were dissolved in DMF (1.0 ml) with stirring under N_2_. Potassium carbonate (304 mg, 2.2 mmol) was added. The reaction mixture was stirred for 24 h at 150 C° and poured into water. The products extracted with CHCl_3_, and washed with brine. The organic layer was dried with MgSO_4_ and concentrated under pressure. Slightly purplish single crystals suitable for X-ray diffraction were obtained by crystallization from ethanol.

### Refinement

All the H atoms were found in difference maps and were subsequently refined as riding atoms, with C—H = 0.95 (aromatic) and 0.98 (methyl) Å, and Uĩso~(H) = 1.2U~eq~(C).

### Crystal data

**Table:**

C_26_H_20_O_4_	*Z* = 2
*M**_r_* = 396.42	*F*_000_ = 416
Triclinic, *P*1	*D*_x_ = 1.363 Mg m^−^^3^
Hall symbol: -P 1	Melting point = 430.2–430.9 K
*a* = 5.8691 (2) Å	Cu *K*α radiation λ = 1.54187 Å
*b* = 7.9105 (2) Å	Cell parameters from 12820 reflections
*c* = 21.4040 (5) Å	θ = 4.2–68.2º
α = 90.322 (2)º	µ = 0.74 mm^−^^1^
β = 95.534 (2)º	*T* = 193 K
γ = 102.283 (2)º	Platelet, clear pale purple
*V* = 966.11 (5) Å^3^	0.60 × 0.20 × 0.02 mm

### Data collection

**Table:**

Rigaku R-AXIS RAPID diffractometer	3467 independent reflections
Radiation source: rotating anode	2617 reflections with *I* > 2σ(*I*)
Monochromator: graphite	*R*_int_ = 0.030
Detector resolution: 10.00 pixels mm^-1^	θ_max_ = 68.2º
*T* = 193 K	θ_min_ = 4.2º
ω scans	*h* = −6→6
Absorption correction: numerical(NUMABS; Higashi, 1999)	*k* = −9→9
*T*_min_ = 0.792, *T*_max_ = 0.985	*l* = −25→25
17040 measured reflections	

### Refinement

**Table:**

Refinement on *F*^2^	Secondary atom site location: difference Fourier map
Least-squares matrix: full	Hydrogen site location: difference Fourier map
*R*[*F*^2^ > 2σ(*F*^2^)] = 0.043	H-atom parameters constrained
*wR*(*F*^2^) = 0.125	*w* = 1/[σ^2^(*F*_o_^2^) + (0.0673*P*)^2^ + 0.0881*P*] where *P* = (*F*_o_^2^ + 2*F*_c_^2^)/3
*S* = 1.09	(Δ/σ)_max_ = 0.001
3467 reflections	Δρ_max_ = 0.19 e Å^−^^3^
273 parameters	Δρ_min_ = −0.24 e Å^−^^3^
Primary atom site location: structure-invariant direct methods	Extinction correction: none

### Special details

**Table:**

Geometry. All e.s.d.'s (except the e.s.d. in the dihedral angle between two l.s. planes) are estimated using the full covariance matrix. The cell e.s.d.'s are taken into account individually in the estimation of e.s.d.'s in distances, angles and torsion angles; correlations between e.s.d.'s in cell parameters are only used when they are defined by crystal symmetry. An approximate (isotropic) treatment of cell e.s.d.'s is used for estimating e.s.d.'s involving l.s. planes.
Refinement. Refinement of *F*^2^ against ALL reflections. The weighted *R*-factor *wR* and goodness of fit *S* are based on *F*^2^, conventional *R*-factors *R* are based on *F*, with *F* set to zero for negative *F*^2^. The threshold expression of *F*^2^ > σ(*F*^2^) is used only for calculating *R*-factors(gt) *etc*. and is not relevant to the choice of reflections for refinement. *R*-factors based on *F*^2^ are statistically about twice as large as those based on *F*, and *R*- factors based on ALL data will be even larger.

### Fractional atomic coordinates and isotropic or equivalent isotropic displacement parameters (Å^2^)

**Table:**

	*x*	*y*	*z*	*U*_iso_*/*U*_eq_	
O1	0.4929 (2)	0.04326 (15)	0.14405 (5)	0.0460 (3)	
O2	0.7532 (2)	0.44294 (17)	−0.10302 (6)	0.0578 (4)	
O3	−0.3337 (2)	0.45641 (15)	0.28446 (5)	0.0445 (3)	
O4	−0.6274 (2)	0.81543 (16)	0.51905 (6)	0.0542 (4)	
C1	0.3180 (3)	0.0478 (2)	0.18309 (7)	0.0364 (4)	
C2	0.2243 (3)	0.1895 (2)	0.19006 (7)	0.0363 (4)	
H2	0.2721	0.2894	0.1661	0.044*	
C3	0.0555 (3)	0.18698 (19)	0.23309 (6)	0.0334 (4)	
C4	−0.0519 (3)	0.3297 (2)	0.24098 (7)	0.0361 (4)	
H4	−0.0100	0.4308	0.2173	0.043*	
C5	−0.2151 (3)	0.3208 (2)	0.28259 (7)	0.0376 (4)	
C6	−0.2807 (3)	0.1748 (2)	0.31916 (7)	0.0416 (4)	
H6	−0.3939	0.1724	0.3482	0.050*	
C7	−0.1796 (3)	0.0366 (2)	0.31236 (7)	0.0402 (4)	
H7	−0.2233	−0.0622	0.3371	0.048*	
C8	−0.0113 (3)	0.03715 (19)	0.26930 (6)	0.0344 (4)	
C9	0.0924 (3)	−0.1059 (2)	0.26025 (7)	0.0410 (4)	
H9	0.0496	−0.2065	0.2841	0.049*	
C10	0.2526 (3)	−0.1022 (2)	0.21793 (7)	0.0407 (4)	
H10	0.3194	−0.1997	0.2120	0.049*	
C11	0.4961 (3)	0.13361 (19)	0.08868 (7)	0.0361 (4)	
C12	0.2975 (3)	0.1256 (2)	0.04757 (7)	0.0402 (4)	
H12	0.1489	0.0664	0.0586	0.048*	
C13	0.3166 (3)	0.2042 (2)	−0.00971 (7)	0.0395 (4)	
H13	0.1800	0.1984	−0.0380	0.047*	
C14	0.5327 (3)	0.29172 (18)	−0.02654 (7)	0.0341 (4)	
C15	0.7292 (3)	0.3012 (2)	0.01629 (7)	0.0393 (4)	
H15	0.8777	0.3623	0.0059	0.047*	
C16	0.7120 (3)	0.2230 (2)	0.07376 (7)	0.0392 (4)	
H16	0.8473	0.2307	0.1027	0.047*	
C17	0.5597 (3)	0.3703 (2)	−0.08936 (7)	0.0403 (4)	
C18	0.3482 (3)	0.3545 (2)	−0.13569 (8)	0.0510 (5)	
H18A	0.3925	0.4167	−0.1736	0.061*	
H18B	0.2301	0.4043	−0.1172	0.061*	
H18C	0.2835	0.2321	−0.1466	0.061*	
C19	−0.3525 (3)	0.52941 (19)	0.34179 (7)	0.0359 (4)	
C20	−0.1817 (3)	0.5418 (2)	0.39204 (7)	0.0404 (4)	
H20	−0.0502	0.4910	0.3891	0.049*	
C21	−0.2046 (3)	0.6289 (2)	0.44668 (7)	0.0409 (4)	
H21	−0.0859	0.6398	0.4808	0.049*	
C22	−0.3988 (3)	0.70055 (19)	0.45228 (7)	0.0359 (4)	
C23	−0.5678 (3)	0.6862 (2)	0.40098 (7)	0.0408 (4)	
H23	−0.7010	0.7351	0.4039	0.049*	
C24	−0.5448 (3)	0.6021 (2)	0.34601 (7)	0.0398 (4)	
H24	−0.6606	0.5942	0.3113	0.048*	
C25	−0.4346 (3)	0.7891 (2)	0.51118 (7)	0.0407 (4)	
C26	−0.2330 (3)	0.8422 (3)	0.56052 (8)	0.0545 (5)	
H26A	−0.2649	0.9313	0.5884	0.065*	
H26B	−0.0902	0.8885	0.5405	0.065*	
H26C	−0.2117	0.7415	0.5851	0.065*	

### Atomic displacement parameters (Å^2^)

**Table:**

	*U*^11^	*U*^22^	*U*^33^	*U*^12^	*U*^13^	*U*^23^
O1	0.0475 (7)	0.0581 (7)	0.0407 (6)	0.0246 (6)	0.0160 (5)	0.0149 (5)
O2	0.0560 (8)	0.0639 (8)	0.0516 (7)	0.0043 (6)	0.0144 (6)	0.0169 (6)
O3	0.0530 (7)	0.0528 (7)	0.0338 (6)	0.0234 (6)	0.0082 (5)	0.0020 (5)
O4	0.0503 (8)	0.0594 (8)	0.0558 (7)	0.0128 (6)	0.0184 (6)	−0.0041 (6)
C1	0.0351 (9)	0.0458 (9)	0.0299 (8)	0.0113 (7)	0.0054 (6)	0.0031 (6)
C2	0.0380 (9)	0.0388 (9)	0.0315 (8)	0.0067 (7)	0.0041 (6)	0.0051 (6)
C3	0.0338 (8)	0.0389 (8)	0.0266 (7)	0.0062 (7)	0.0010 (6)	0.0007 (6)
C4	0.0395 (9)	0.0370 (8)	0.0311 (8)	0.0064 (7)	0.0038 (6)	0.0030 (6)
C5	0.0385 (9)	0.0427 (9)	0.0332 (8)	0.0127 (7)	0.0025 (6)	−0.0026 (6)
C6	0.0407 (10)	0.0492 (10)	0.0352 (8)	0.0070 (8)	0.0117 (7)	0.0014 (7)
C7	0.0418 (10)	0.0405 (9)	0.0364 (8)	0.0029 (7)	0.0073 (7)	0.0050 (7)
C8	0.0338 (9)	0.0388 (8)	0.0294 (7)	0.0054 (7)	0.0024 (6)	0.0014 (6)
C9	0.0466 (10)	0.0372 (9)	0.0383 (8)	0.0063 (7)	0.0058 (7)	0.0059 (7)
C10	0.0464 (10)	0.0401 (9)	0.0386 (9)	0.0151 (7)	0.0063 (7)	0.0032 (7)
C11	0.0411 (9)	0.0378 (8)	0.0328 (8)	0.0136 (7)	0.0087 (7)	0.0033 (6)
C12	0.0335 (9)	0.0448 (9)	0.0413 (9)	0.0034 (7)	0.0099 (7)	0.0014 (7)
C13	0.0352 (9)	0.0461 (9)	0.0363 (8)	0.0075 (7)	0.0021 (7)	−0.0014 (7)
C14	0.0375 (9)	0.0310 (8)	0.0349 (8)	0.0085 (6)	0.0064 (6)	−0.0014 (6)
C15	0.0339 (9)	0.0390 (9)	0.0438 (9)	0.0031 (7)	0.0085 (7)	0.0023 (7)
C16	0.0341 (9)	0.0466 (9)	0.0375 (8)	0.0106 (7)	0.0025 (7)	0.0004 (7)
C17	0.0483 (10)	0.0364 (8)	0.0390 (9)	0.0126 (7)	0.0103 (7)	0.0020 (7)
C18	0.0608 (12)	0.0583 (11)	0.0380 (9)	0.0221 (9)	0.0049 (8)	0.0060 (8)
C19	0.0377 (9)	0.0370 (8)	0.0346 (8)	0.0083 (7)	0.0103 (7)	0.0032 (6)
C20	0.0353 (9)	0.0462 (9)	0.0426 (9)	0.0143 (7)	0.0053 (7)	0.0019 (7)
C21	0.0388 (9)	0.0459 (9)	0.0390 (9)	0.0123 (7)	0.0019 (7)	0.0027 (7)
C22	0.0358 (9)	0.0335 (8)	0.0383 (8)	0.0053 (6)	0.0079 (7)	0.0031 (6)
C23	0.0332 (9)	0.0433 (9)	0.0478 (9)	0.0103 (7)	0.0081 (7)	0.0022 (7)
C24	0.0345 (9)	0.0446 (9)	0.0411 (9)	0.0110 (7)	0.0020 (7)	0.0016 (7)
C25	0.0445 (10)	0.0369 (8)	0.0418 (9)	0.0073 (7)	0.0129 (7)	0.0054 (7)
C26	0.0574 (12)	0.0635 (12)	0.0432 (10)	0.0149 (9)	0.0048 (8)	−0.0075 (8)

### Geometric parameters (Å, °)

**Table:**

O1—C11	1.3871 (17)	C13—C14	1.391 (2)
O1—C1	1.3905 (17)	C13—H13	0.9500
O2—C17	1.2225 (18)	C14—C15	1.391 (2)
O3—C19	1.3772 (17)	C14—C17	1.492 (2)
O3—C5	1.4001 (18)	C15—C16	1.383 (2)
O4—C25	1.2201 (19)	C15—H15	0.9500
C1—C2	1.363 (2)	C16—H16	0.9500
C1—C10	1.408 (2)	C17—C18	1.495 (2)
C2—C3	1.413 (2)	C18—H18A	0.9800
C2—H2	0.9500	C18—H18B	0.9800
C3—C4	1.422 (2)	C18—H18C	0.9800
C3—C8	1.425 (2)	C19—C24	1.381 (2)
C4—C5	1.361 (2)	C19—C20	1.386 (2)
C4—H4	0.9500	C20—C21	1.386 (2)
C5—C6	1.405 (2)	C20—H20	0.9500
C6—C7	1.363 (2)	C21—C22	1.391 (2)
C6—H6	0.9500	C21—H21	0.9500
C7—C8	1.414 (2)	C22—C23	1.394 (2)
C7—H7	0.9500	C22—C25	1.493 (2)
C8—C9	1.415 (2)	C23—C24	1.381 (2)
C9—C10	1.363 (2)	C23—H23	0.9500
C9—H9	0.9500	C24—H24	0.9500
C10—H10	0.9500	C25—C26	1.495 (2)
C11—C16	1.381 (2)	C26—H26A	0.9800
C11—C12	1.381 (2)	C26—H26B	0.9800
C12—C13	1.381 (2)	C26—H26C	0.9800
C12—H12	0.9500		
			
C11—O1—C1	119.52 (12)	C13—C14—C17	122.02 (14)
C19—O3—C5	118.88 (11)	C16—C15—C14	121.10 (14)
C2—C1—O1	123.00 (14)	C16—C15—H15	119.5
C2—C1—C10	121.89 (14)	C14—C15—H15	119.5
O1—C1—C10	115.03 (14)	C11—C16—C15	119.34 (15)
C1—C2—C3	119.57 (14)	C11—C16—H16	120.3
C1—C2—H2	120.2	C15—C16—H16	120.3
C3—C2—H2	120.2	O2—C17—C14	120.20 (15)
C2—C3—C4	121.79 (14)	O2—C17—C18	120.69 (14)
C2—C3—C8	119.44 (14)	C14—C17—C18	119.09 (14)
C4—C3—C8	118.76 (13)	C17—C18—H18A	109.5
C5—C4—C3	119.74 (14)	C17—C18—H18B	109.5
C5—C4—H4	120.1	H18A—C18—H18B	109.5
C3—C4—H4	120.1	C17—C18—H18C	109.5
C4—C5—O3	117.97 (14)	H18A—C18—H18C	109.5
C4—C5—C6	122.15 (15)	H18B—C18—H18C	109.5
O3—C5—C6	119.65 (14)	O3—C19—C24	116.52 (14)
C7—C6—C5	119.04 (14)	O3—C19—C20	122.80 (14)
C7—C6—H6	120.5	C24—C19—C20	120.53 (14)
C5—C6—H6	120.5	C19—C20—C21	119.39 (15)
C6—C7—C8	121.46 (14)	C19—C20—H20	120.3
C6—C7—H7	119.3	C21—C20—H20	120.3
C8—C7—H7	119.3	C20—C21—C22	121.00 (15)
C7—C8—C9	122.66 (14)	C20—C21—H21	119.5
C7—C8—C3	118.84 (14)	C22—C21—H21	119.5
C9—C8—C3	118.50 (14)	C21—C22—C23	118.36 (14)
C10—C9—C8	121.31 (14)	C21—C22—C25	122.64 (15)
C10—C9—H9	119.3	C23—C22—C25	118.98 (14)
C8—C9—H9	119.3	C24—C23—C22	121.06 (15)
C9—C10—C1	119.28 (15)	C24—C23—H23	119.5
C9—C10—H10	120.4	C22—C23—H23	119.5
C1—C10—H10	120.4	C23—C24—C19	119.63 (15)
C16—C11—C12	120.70 (14)	C23—C24—H24	120.2
C16—C11—O1	116.81 (14)	C19—C24—H24	120.2
C12—C11—O1	122.32 (14)	O4—C25—C22	120.09 (15)
C11—C12—C13	119.49 (14)	O4—C25—C26	120.68 (15)
C11—C12—H12	120.3	C22—C25—C26	119.22 (14)
C13—C12—H12	120.3	C25—C26—H26A	109.5
C12—C13—C14	121.00 (15)	C25—C26—H26B	109.5
C12—C13—H13	119.5	H26A—C26—H26B	109.5
C14—C13—H13	119.5	C25—C26—H26C	109.5
C15—C14—C13	118.34 (14)	H26A—C26—H26C	109.5
C15—C14—C17	119.62 (14)	H26B—C26—H26C	109.5
			
C11—O1—C1—C2	−34.0 (2)	O1—C11—C12—C13	−173.43 (14)
C11—O1—C1—C10	149.36 (14)	C11—C12—C13—C14	−0.2 (2)
O1—C1—C2—C3	−176.78 (13)	C12—C13—C14—C15	−1.2 (2)
C10—C1—C2—C3	−0.4 (2)	C12—C13—C14—C17	176.96 (14)
C1—C2—C3—C4	−178.38 (14)	C13—C14—C15—C16	1.2 (2)
C1—C2—C3—C8	1.0 (2)	C17—C14—C15—C16	−177.04 (14)
C2—C3—C4—C5	179.42 (14)	C12—C11—C16—C15	−1.7 (2)
C8—C3—C4—C5	0.0 (2)	O1—C11—C16—C15	173.65 (13)
C3—C4—C5—O3	−173.71 (12)	C14—C15—C16—C11	0.3 (2)
C3—C4—C5—C6	0.7 (2)	C15—C14—C17—O2	−0.2 (2)
C19—O3—C5—C4	−132.00 (15)	C13—C14—C17—O2	−178.38 (14)
C19—O3—C5—C6	53.40 (19)	C15—C14—C17—C18	178.13 (14)
C4—C5—C6—C7	−0.7 (2)	C13—C14—C17—C18	0.0 (2)
O3—C5—C6—C7	173.69 (14)	C5—O3—C19—C24	−152.32 (14)
C5—C6—C7—C8	−0.2 (2)	C5—O3—C19—C20	32.1 (2)
C6—C7—C8—C9	−178.51 (14)	O3—C19—C20—C21	175.05 (14)
C6—C7—C8—C3	0.9 (2)	C24—C19—C20—C21	−0.4 (2)
C2—C3—C8—C7	179.76 (13)	C19—C20—C21—C22	1.4 (2)
C4—C3—C8—C7	−0.8 (2)	C20—C21—C22—C23	−1.5 (2)
C2—C3—C8—C9	−0.8 (2)	C20—C21—C22—C25	177.50 (14)
C4—C3—C8—C9	178.62 (13)	C21—C22—C23—C24	0.4 (2)
C7—C8—C9—C10	179.34 (14)	C25—C22—C23—C24	−178.56 (14)
C3—C8—C9—C10	−0.1 (2)	C22—C23—C24—C19	0.6 (2)
C8—C9—C10—C1	0.7 (2)	O3—C19—C24—C23	−176.32 (13)
C2—C1—C10—C9	−0.5 (2)	C20—C19—C24—C23	−0.6 (2)
O1—C1—C10—C9	176.16 (13)	C21—C22—C25—O4	−163.64 (15)
C1—O1—C11—C16	137.94 (14)	C23—C22—C25—O4	15.3 (2)
C1—O1—C11—C12	−46.8 (2)	C21—C22—C25—C26	15.4 (2)
C16—C11—C12—C13	1.7 (2)	C23—C22—C25—C26	−165.70 (15)

### Hydrogen-bond geometry (Å, °)

**Table:**

*D*—H···*A*	*D*—H	H···*A*	*D*···*A*	*D*—H···*A*
C2—H2···O2^i^	0.95	2.54	3.448 (2)	160

Symmetry codes: (i) −*x*+1, −*y*+1, −*z*.
